# The effect of muscle length on transcranial magnetic stimulation‐induced relaxation rate in the plantar flexors

**DOI:** 10.14814/phy2.13442

**Published:** 2017-09-26

**Authors:** Alexandra F. Yacyshyn, Jane Nettleton, Geoffrey A. Power, Jennifer M. Jakobi, Chris J. McNeil

**Affiliations:** ^1^ School of Health and Exercise Sciences University of British Columbia Kelowna British Columbia Canada; ^2^ Centre for Heart, Lung and Vascular Health Faculty of Health and Social Development University of British Columbia Kelowna British Columbia Canada; ^3^ Department of Human Health & Nutritional Sciences College of Biological Sciences University of Guelph Guelph Ontario Canada; ^4^ Healthy Exercise and Aging Laboratory Group Faculty of Health and Social Development University of British Columbia Kelowna British Columbia Canada

**Keywords:** Medial gastrocnemius, muscle fascicles, muscle–tendon junction, silent period, ultrasonography

## Abstract

Transcranial magnetic stimulation (TMS) of the motor cortex during a maximal voluntary contraction (MVC) permits functionally relevant measurements of muscle group relaxation rate (i.e., when muscles are actively contracting under voluntary control). This study's purpose was twofold: (1) to explore the impact of muscle length on TMS‐induced plantar flexor relaxation rate; and (2) to incorporate ultrasonography to measure relaxation‐induced lengthening of medial gastrocnemius (MG) fascicles and displacement of the muscle–tendon junction (MTJ). Eleven males (24.8 ± 7.0 years) performed 21 brief isometric plantar flexor MVCs. Trials were block‐randomized every three MVCs among 20° dorsiflexion (DF), a neutral ankle position, and 30° plantar flexion (PF). During each MVC, TMS was delivered and ultrasound video recordings captured MG fascicles or MTJ length changes. Peak relaxation rate was calculated as the steepest slope of the TMS‐induced drop in plantar flexor torque or the rate of length change for MG fascicles and MTJ. Torque relaxation rate was slower for PF (−804 ± 162 Nm·s^−1^) than neutral and DF (−1896 ± 298 and −2008 ± 692 Nm·s^−1^, respectively). Similarly, MG fascicle relaxation rate was slower for PF (−2.80 ± 1.10 cm·s^−1^) than neutral and DF (−5.35 ± 1.10 and −4.81 ± 1.87 cm·s^−1^, respectively). MTJ displacement rate showed a similar trend (*P* = 0.06), with 3.89 ± 1.93 cm·s^−1^ for PF compared to rates of 6.87 ± 1.55 and 6.36 ± 2.97 cm·s^−1^ for neutral and DF, respectively. These findings indicate muscle length affects the torque relaxation rate recorded after TMS during an MVC. Comparable results were obtained from muscle fascicles, indicating ultrasound imaging is suitable for measuring evoked contractile properties during voluntary contraction.

## Introduction

Voluntary force production is dependent on both central and peripheral elements working in concert within the neuromuscular system (Bigland‐Ritchie et al. 1983; Gandevia 2001). The development of noninvasive stimulation techniques has allowed researchers to probe intrinsic muscle contractile properties to better understand the mechanisms underlying the production of force. While electrical stimulation of a peripheral nerve or intramuscular nerve fibers during a relaxed muscle state is the standard technique for the investigation of intrinsic contractile properties, transcranial magnetic stimulation (TMS) during a maximal voluntary contraction (MVC) (e.g., Todd et al. 2005, 2007) has numerous advantages for the assessment of muscle relaxation. These advantages have been identified (Todd et al. 2007; McNeil et al. 2013) but the principal benefit of TMS during an MVC over electrical stimulation of a relaxed muscle is the functional relevance; that is, the measurement of relaxation when the central nervous system is actively driving the muscle. It is well established that muscle properties are dependent on the contractile state and this is clear when one considers the marked age‐related slowing of elbow flexor relaxation rate shown with TMS (Hunter et al. 2008; Molenaar et al. 2013) which was not observed with an electrically induced twitch response in relaxed muscle (Doherty et al. 1993; Allman and Rice 2001; Dalton et al. 2010).

In addition to being able to detect age‐related changes to the neuromuscular system, the technique of TMS‐induced relaxation has also shown the ability to detect differences based on sex (Hunter et al. 2006), temperature (Todd et al. 2005), fatigue (Todd et al. 2005, 2007) as well as stimulus intensity and relative contribution of synergist muscles (McNeil et al. 2013). Relaxation rate of the plantar flexor muscle group was significantly slower with the knee flexed compared to extended (McNeil et al. 2013). The change was ascribed to a greater relative contribution of the predominantly slow‐twitch soleus when the biarticulate, mixed‐fiber gastrocnemii were placed in a shortened position, thus diminishing the relative contribution of the gastrocnemii to overall torque production. However, as the absolute contribution of the soleus (and all other synergists, aside from the plantaris) was constant at the two knee angles, the results of this study (McNeil et al. 2013) do not indicate whether relaxation rates induced by TMS will differ with changes in the length–tension relationship of the entire plantar flexor muscle group. Given the potential clinical utility of the TMS‐induced relaxation technique (Kleine & Stegeman 2007), and the dependence of relaxation properties of a resting twitch on muscle length (e.g., Hartree & Hill 1921; Sale et al. 1982), it is important to investigate the impact that muscle length has on relaxation rates measured with this technique. Manipulation of the joint common to all synergists (i.e., the ankle joint in the case of the plantar flexor muscles) will permit this assessment.

To date, the TMS‐induced relaxation technique has focused solely on the torque response of a muscle group. Hence, it is unknown whether the muscle group relaxation rate is reflected at the level of muscle fascicles and/or the muscle–tendon junction (MTJ). Advancements in ultrasonography make the investigation of these components possible as improved resolution and sampling rates have led to the measurement of in vivo architectural changes during voluntary isometric contractions (e.g., Fukunaga et al. 1997; Ito et al. 1998; Maganaris 2003; Arampatzis et al. 2007; Herbert et al. 2011; Power et al. 2013). However, only a small number of studies have used ultrasonography to investigate intrinsic contractile properties (i.e., length–tension relationship) and all of these involved electrical stimulation of a relaxed muscle (Maganaris and Paul 2000; Gao and Zhang 2008; Nordez et al. 2009; Mayfield et al. 2016a). Hence, no study has paired TMS with ultrasound to assess intrinsic contractile properties while the muscle is actively contracting.

The first objective of this study was to test the impact of muscle length on relaxation induced by TMS via a comparison of plantar flexor torque relaxation rates at three ankle angles. The second objective was to determine if the relaxation rates observed at the level of the plantar flexor muscle group were also reflected at the level of muscle fascicles and the MTJ for the medial gastrocnemius (MG), as determined via ultrasonography. It was hypothesized that muscle length would affect plantar flexor relaxation rate following TMS and that the relaxation rate of MG fascicles and the rate of MTJ displacement would show a similar impact of joint angle.

## Methods

### Subjects

Thirteen healthy male participants were recruited for this study; they gave written informed consent prior to testing and all experimental designs were approved by the institutional research ethics committee. Participants had no indication of neuromuscular or cardiovascular pathologies and no contraindications to TMS. To ensure that a large superimposed twitch did not influence the maximal relaxation rate, participants were required to achieve an estimated voluntary activation value of >90% throughout the protocol. Two participants were excluded for low voluntary activation values in the dorsiflexed (DF) position (~85%); therefore, all data reported were collected from the remaining eleven participants (age 24.8 ± 7.0 years, height 179.2 ± 9.4 cm, mass 83.6 ± 14.1 kg, tibial length 41.6 ± 3.3 cm; mean ± SD). Torque data are *n* = 11, MG fascicle data are *n* = 10 (one subject was excluded due to extreme fascicle curvature), and MTJ data are *n* = 8 (two additional subjects were excluded as the probe was displaced by conformational changes of the musculature during contraction).

### Participant set‐up

Participants lay prone on a HUMAC NORM dynamometer (CSMi, Stoughton, MA) with their right knee extended fully and their right foot firmly secured to the dynamometer footplate. All participants were right leg dominant. Three ankle angles were tested for all components of this study: 20° DF, a neutral ankle position (90° between the tibia and the plantar aspect of the foot), and 30° plantar flexion (PF). Plantar flexor torque was measured and electromyographic activity (EMG) of the lateral gastrocnemius was recorded via adhesive Ag‐AgCl electrodes (10 mm diameter) positioned 2 cm apart over the muscle belly. A ground electrode was positioned over the patella. Torque and EMG signals were sampled online at 1000 and 2000 Hz, respectively, using a 16‐bit A/D converter (CED Power 1401‐3; Cambridge Electronics Design, Cambridge, UK) in combination with Spike2 software (v. 7.10; Cambridge Electronic Design). EMG data were amplified (100×) and bandpass filtered (16–1000 Hz) using CED 1902 amplifiers (Cambridge Electronic Design).

### Transcranial magnetic stimulation

TMS was delivered to the motor cortex using a double‐cone coil (13 cm outside diameter for each cone) attached via a BiStim^2^ unit to two Magstim 200^2^ stimulators (Magstim, Whitland, UK). The direction of current flow in the coil preferentially activated the left hemisphere. With the participant performing a weak submaximal plantar flexion contraction, optimal coil placement was determined by delivering single stimuli (at 40% of stimulator output) every 5–10 sec as coil position was systematically adjusted (1 cm increments in the lateral then ventral–dorsal directions). The coil position yielding the largest motor evoked potential (MEP) in the lateral gastrocnemius (~1 cm left of the vertex) was indicated with an indelible marker on the subject's scalp for use throughout the remainder of the experiment.

Participants were gradually familiarized to high‐intensity TMS with the following isometric contraction‐TMS intensity pairings: 10% MVC at 40% TMS, 10% MVC at 80% TMS, 50% MVC at 80% TMS, and 100% MVC at 80% TMS. It has been shown previously that a stimulus intensity of 60% induces a plantar flexor relaxation rate which is equivalent to a relaxation rate at 100% stimulator output, and is more pleasant for the participant (McNeil et al. 2013). An intensity of 80% stimulator output was selected to ensure a maximal relaxation rate was achieved.

### Ultrasound imaging

Given the soleus presents as hypoechoic during an MVC (impairing visibility for fascicle measures) and the lateral gastrocnemius fascicles are considerably longer than the MG (requiring additional extrapolation to measure complete lengths; see Kawakami et al. 1998), the MG was chosen for ultrasound analysis. While the MG can act as a proxy for the triceps surae muscles, architectural measurements should be generalized with prudence, due to contractile kinetics differences between individual plantar flexors (Vandervoort and McComas 1983).

All ultrasound images and videos were recorded with real‐time B‐mode ultrasonography (GE LOGIQ E9, Connecticut) using a ML6‐15‐D transducer (15.0 MHz linear array, 50 mm field‐of‐view). The transducer was placed in parallel with the structure of interest (along the median longitudinal axis) for all measurements; small adjustments around the proximodistal and anteroposterior axes were made prior to each recording to ensure the clearest image of the muscle fascicles was obtained (Herbert et al. 2011).

#### Imaging at rest

Still frame images (for MTJ length as well as the length and pennation angle of MG muscle fascicles) and LOGIQview scans (for Achilles tendon (AT) length and MG muscle belly length) were taken with the participant at rest. Depending on muscle size, the depth of field ranged from 2.8 to 4.0 cm. The position and orientation of the transducer was manually controlled by the operator during each image/scan.

The location of the MTJ was identified as the point at which the MG and AT merged; probe position was marked on the skin with indelible marker to ensure consistent placement. An anechoic marker was used to allow for measurement comparisons among ankle angles. Muscle fascicle images were taken over the center of the MG muscle belly; as with the MTJ, probe position was marked on the skin for consistency. AT length was scanned from the insertion onto the calcaneus to the MTJ (Zhao et al. 2009). MG belly length was scanned from the MTJ to the popliteal crease and normalized to tibial length.

#### Imaging during MVCs

During the 21 MVCs of the main protocol, a video recording (77 frames‐per‐second and 2.8 cm depth of field) was taken of MG fascicles or the MTJ. A custom‐made foam brace with inelastic straps was used to secure the probe during contractions; if small transducer shifts were noted, the probe was returned to the position denoted by markings on the skin.

### Experimental procedures

Data collection began with the determination of tibial length, as measured from the medial malleolus to medial condyle with a flexible tape measure. Following this, participants were positioned on the dynamometer and ultrasound images were collected at rest for AT length, MG belly length, MTJ excursion from the neutral position, and MG fascicle lengths and pennation angles. Three images were taken at each ankle angle (20° DF, followed by neutral and 30° PF) for every variable.

Participants were then positioned at a neutral ankle angle for optimal TMS coil placement, as described above. This was followed by 2–3 practice MVCs with TMS at 80% of the stimulator output, to ensure participants were familiar with the procedure prior to data collection for the main experimental protocol. Strong verbal encouragement and visual feedback of plantar flexor torque were given during all MVCs; participants were instructed to plantar flex “hard and fast” and continue contracting maximally following the click of the TMS. At least 90 sec of rest separated each contraction.

**Figure 1:**
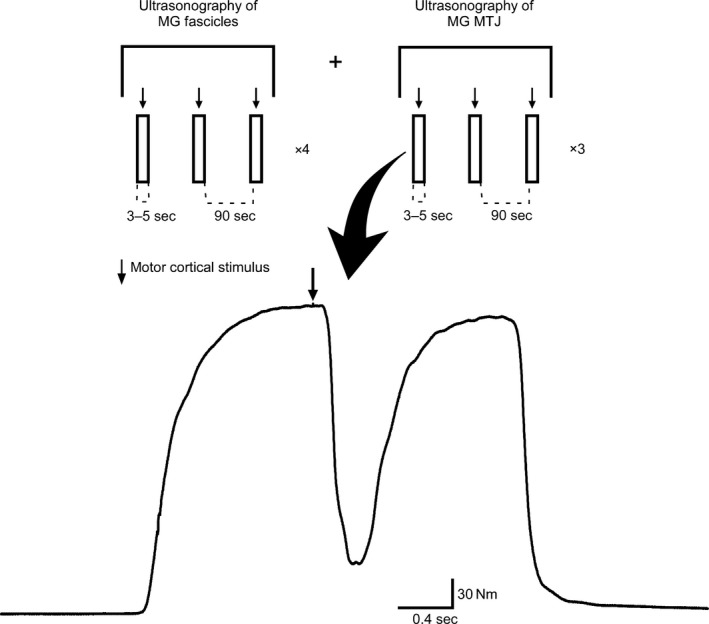
Protocol schematic. (Top) Each rectangle represents one brief (3 sec) MVC paired with TMS. A total of 21 MVCs were performed and a minimum of 90 sec rest was provided between contractions. One ultrasound video recording was taken per MVC, with 12 consecutive recordings of MG fascicles and nine consecutive recordings of MG muscle–tendon junction (MTJ). (Bottom) Raw trace of plantar flexor torque during a DF MVC, showcasing TMS‐induced relaxation (arrow denotes the timing of TMS delivery).

The experimental protocol involved a total of 21 brief (~3 sec) isometric plantar flexor MVCs in blocks of three MVCs. Within each block, participants performed one MVC at each ankle angle (20° DF, neutral, and 30° PF) in a randomized order. Specifically, ankle angle order was randomized within blocks prior to study initiation and all participants completed the same prerandomized set of blocks. During each MVC, TMS was delivered to the motor cortex (Fig. 1). Ultrasound video recordings were captured over the MG muscle belly (12 consecutive MVCs) and MTJ (nine consecutive MVCs), with the initial imaging location randomized for all participants. Video recordings were started immediately before the participant was prompted to plantar flex and stopped after the MVC was completed. A minimum of 90 sec rest was given between each MVC and approximately 3 min of rest was given when the imaging site was changed.

### Data analysis

#### Torque

Signal software (v. 5.08; Cambridge Electronic Design) was used offline to analyze torque. Mean torque was calculated over 100 msec (in the interval 250 to 150 msec prior to TMS) to obtain a prestimulus measure of total torque. As previously described (Taylor et al. 2000; Todd et al. 2003), voluntary activation was estimated using the equation: voluntary activation (%) = (1 – [superimposed twitch torque/MVC torque prior to TMS]) × 100. Passive torque was determined as the value of the baseline prior to the start of the MVC; in the results, passive torque values are normalized such that at the PF ankle angle they equal zero. Active torque was calculated as total torque minus passive torque. Peak relaxation rate for the plantar flexors was measured as the steepest negative torque slope over a 10 msec interval (5 msec on each side of the steepest instantaneous slope; McNeil et al. 2013; Molenaar et al. 2013). To eliminate high‐frequency noise which led to erroneous identification of the steepest instantaneous slope, a three‐point smoothing feature was applied to torque data for the analysis of relaxation rate. Normalized plantar flexor relaxation rate was calculated by dividing the peak plantar flexor relaxation rate by the prestimulus torque (as measured 5 msec before TMS delivery).

#### Ultrasonography: General

**Figure 2:**
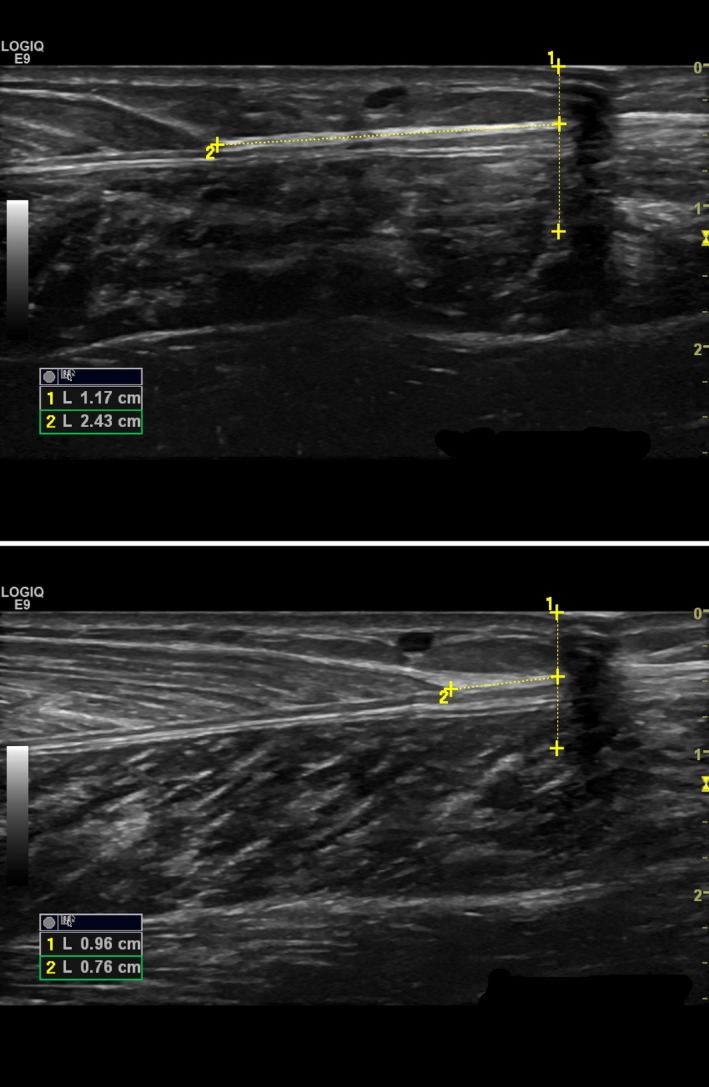
Video recording still image captures of muscle–tendon junction length change (1.67 cm in 0.27 sec) for a single participant during TMS‐induced relaxation at a neutral (0°) ankle angle, from MVC (top) to the moment of maximal TMS‐induced relaxation (bottom).

Ultrasound images were analyzed offline using the GE LOGIQ E9 software inherent to the machine. AT length was measured using an open spline trace from the MTJ to the insertion point on the calcaneus. MG belly length was measured from the MTJ to the proximal edge of the scan (deliberately stopped at the popliteal crease) with a straight‐line function. MTJ displacement from neutral to DF or PF positions (passive) and from MVC to TMS‐induced relaxation (active) was determined using a straight‐line function from the MTJ to the shadow cast by a static, anechoic marker (Fig. 2). The change in MTJ length was averaged for each ankle angle.

**Figure 3:**
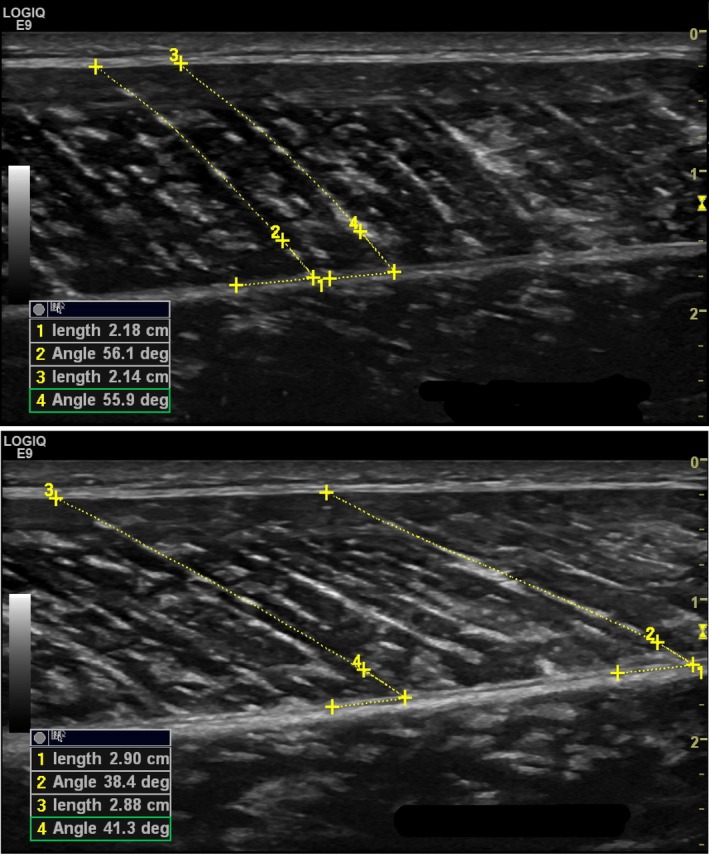
Video recording still image captures of MG muscle fascicle changes in length and pennation angle for a single participant during TMS‐induced relaxation (duration of 0.30 sec) at a plantar flexed (30°) ankle angle, from MVC (top) to the moment of maximal TMS‐induced relaxation (bottom).

Muscle fascicle length was measured from the lower to the upper aponeurosis with an open spline trace (Fig. 3), enabling adjustments for nonlinearity (i.e., fascicle curvature). The Law of Cosines was used to extrapolate the length of straight muscle fascicles if the top portion of the fascicle (joining the upper aponeurosis) shifted outside the field‐of‐view (Reeves et al. 2004; Power et al. 2013). Extrapolation was employed for approximately 30% of all measured fascicles. An inherent angle function was used to measure pennation angle at the point where the muscle fascicle joined the lower aponeurosis.

#### Ultrasonography: Relaxation rates

From each MVC with the probe over the MG belly (four at each angle), the lengths of two fascicles were measured from the frame prior to visible fascicle lengthening (start of TMS‐induced relaxation) and two additional fascicles were measured from the frame prior to the redevelopment of active tension (end of TMS‐induced relaxation). To obtain a change in fascicle length at each ankle angle, the mean postrelaxation length was subtracted from the mean prerelaxation length. For each MVC, the fascicle frame numbers were noted and the difference of the pre‐ and postrelaxation values was divided by the video frame rate (77 frames‐per‐second) to give the time‐to‐relaxation. The time‐to‐relaxation was then averaged for the four MVCs at each ankle angle to obtain a mean value of time elapsed for TMS‐induced fascicle relaxation to occur. The rate of fascicle relaxation at each angle was calculated using the following equation: relaxation rate (cm·s^−1^) = mean change in fascicle length ÷ mean time‐to‐relaxation.

Fascicle data were averaged as described because single fascicles could not (with certainty) be visually tracked over the rapid period of TMS‐induced relaxation. Despite measures taken to secure probe placement, there was minute movement relative to the participant as a result of the abrupt relaxation induced by TMS. We compensated for the inability to track single fascicles through an MVC by having participants perform four contractions at each ankle angle and then averaging values within a participant.

In contrast to single fascicles, the MTJ could be tracked through the TMS‐induced relaxation so it was unnecessary to average pre‐ and postrelaxation lengths across MVCs. Instead, for each MVC, the rate of MTJ displacement was calculated as the *change in length ÷ time to relaxation*. The mean at each angle was then taken as the average of the three MVCs.

### Statistics

Using SPSS (version 22.0, SPSS Inc., Chicago, IL), separate one‐way repeated measures ANOVAs were run to test the effect of ankle angle (DF, neutral, and PF) on passive torque, active torque, absolute plantar flexor relaxation rate, and normalized plantar flexor relaxation rate. A mixed linear model was used to assess the relationship between passive torque and absolute plantar flexor relaxation rate. Additionally, a paired‐samples t‐test of mean MVC torque for the first three contractions compared to the last three contractions was run to determine whether fatigue developed over the course of the protocol.

Separate one‐way repeated measures ANOVAs were run to test the effect of ankle angle for MG and AT length at rest. The impacts of ankle angle and muscle state (rest, during MVC, and TMS‐induced relaxation) were assessed with two univariate ANOVAs, one for MG fascicle length and another for pennation angle. The effect of ankle angle was assessed for MG fascicle relaxation rates with a univariate one‐factor ANOVA. Finally, the effect of ankle angle on the rate of MTJ displacement was tested with an one‐way repeated measures ANOVA. Main effects of ankle angle or muscle state were followed by post hoc testing, for which a Bonferroni correction factor was applied to multiple comparisons.

Intrarater reliability for ultrasound measurements was determined using mean MG fascicle length data from two participants (total number of fascicles reanalyzed, *n* = 131). These data were analyzed on two occasions (the initial analysis compared to a reanalysis ~4 months later by the same experimenter) and tested with a two‐way mixed, absolute agreement intraclass correlation coefficient (ICC). The ICC value was given as 0.998 (*P* < 0.001), indicating excellent intrarater reliability. Significance for all data was defined as *P* < 0.05. Data are presented as the mean value ± standard deviation (SD) for tables and mean ± standard error of the mean (SEM) for figures.

## Results

TMS‐induced absolute relaxation rates for the plantar flexor muscles and MG muscle fascicles are significantly slower (40–60%) for shortened muscle lengths (PF) compared to longer muscle lengths (DF and neutral) (*P* < 0.05; see text for more details). The rate of MTJ displacement during relaxation follows the same trend (*P* = 0.06).

### Plantar flexor muscle group data

Passive and active torque were normalized such that the passive torque in the PF position equaled zero. Passive torque was shown to be significantly greater (*P* < 0.001) for DF (18.8 ± 6.9 Nm) compared to neutral (5.8 ± 2.1 Nm) and PF, and for neutral compared to PF. Similarly, active torque was greater for DF (182.2 ± 39.8 Nm) compared to neutral (147.8 ± 18.7 Nm; *P* = 0.001) and PF (62.2 ± 9.6 Nm; *P* < 0.001), and for neutral compared to PF (*P* < 0.001). Estimated voluntary activation was high for all participants at each ankle angle (97.3 ± 1.6, 97.9 ± 1.5 and 97.2 ± 2.1% for DF, neutral and PF, respectively). A paired‐samples t‐test of mean MVC torque showed no significant difference (*P* = 0.354) between the first three contractions (133.7 ± 27.8 Nm) compared to the last three contractions (127.9 ± 19.3 Nm), indicating that fatigue did not develop during the course of the protocol.

Absolute plantar flexor relaxation rates (as determined from the TMS‐induced drop in MVC torque) were not significantly different between DF and neutral positions (*P* = 1.000; −2008 ± 692 Nm·s^−1^ and −1896 ± 298 Nm·s^−1^, respectively); however, both DF and neutral positions had significantly faster peak relaxation rates compared to PF (−804 ± 162 Nm·s^−1^; *P* < 0.001). In contrast to absolute rates, normalized plantar flexor relaxation rates were not different across ankle angles (*F*(1,10) = 1.18; *P* = 0.312; −11.1 ± 3.0 s^−1^ DF, −13.1 ± 2.3 s^−1^ neutral, and −13.4 ± 2.3 s^−1^ PF).

There was a moderate relationship (*r* = 0.493, *P* ≤ 0.001) between passive torque and plantar flexor relaxation rate, indicating that higher passive tensions, associated with longer muscle lengths, were correlated with faster absolute rates of muscle relaxation.

### Ultrasound data

#### MG and AT lengths at rest

Absolute and normalized (as percent of tibial length) MG length significantly decreased from the DF position to the PF position (*P* < 0.001; see Table 1). Additionally, absolute AT length showed significant shortening from DF to neutral (*P* = 0.004) and from DF to PF (*P* = 0.016), but no significant difference between neutral and PF (*P* = 1.000; see Table 1).

**Table 1:** Normalized MG length, absolute MG length, and absolute AT length for three ankle angles

Measurement	DF	Neutral	PF
Normalized MG length (% tibial length)	54.05 ± 4.12*	51.66 ± 3.90	48.26 ± 4.16^†^
MG length (cm)	22.38 ± 2.51*	21.39 ± 2.33	19.98 ± 2.28^†^
AT length (cm)	22.30 ± 2.43*^‡^	22.02 ± 2.34	21.91 ± 2.41

Values are presented as mean ± SD; *n* = 10.

*P* < 0.05 compared to ankle angle at: neutral (*); DF and neutral (†); and PF (‡).

MG, medial gastrocnemius; AT, Achilles tendon; DF, dorsiflexion; PF, plantar flexion.

#### Fascicles lengths and pennation angles

Ankle angle and muscle state had a significant interaction (*F*(4,72) = 4.572; *P* = 0.002) and main effects (*F*(2,72) = 156.159; *P* < 0.001 and *F*(2,72) = 286.549; *P* < 0.001, respectively) for MG fascicle length. Likewise, ankle angle and muscle state had a significant interaction (*F*(4,72) = 7.370; *P* < 0.001) and main effects (*F*(2,72) = 219.447; *P* < 0.001 and *F*(2.72) = 88.708; *P* < 0.001, respectively) for pennation angle. Post hoc analyses showed that all ankle angles differed from one another for MG fascicle length (DF > neutral > PF) and pennation angle (DF < neutral < PF) (all *P* < 0.001; Table 2). Similarly, each muscle state differed from the other two for MG fascicle length (rest > TMS‐induced relaxation > MVC) and pennation angle (rest < TMS‐induced relaxation < MVC) (all *P* < 0.001; Table 2).

**Table 2:** MG fascicle length and pennation angle for three ankle angles over three muscle states

Measurement & muscle state	DF	Neutral	PF
Fascicle length (cm)			
At rest	7.28 ± 1.52*	6.35 ± 1.28	4.66 ± 0.95^†^
During MVC	4.10 ± 0.93*^§^	3.06 ± 0.56^§^	2.51 ± 0.42^†§^
TMS‐induced relaxation	5.50 ± 1.07*^#^	4.52 ± 0.75^#^	3.31 ± 0.59^†#^
Pennation angle (°)			
At rest	22.49 ± 2.52*	23.91 ± 3.28	28.45 ± 4.21^†^
During MVC	35.68 ± 6.84*^§^	43.80 ± 7.42^§^	53.69 ± 7.90^†§^
TMS‐induced relaxation	28.73 ± 4.18*^#^	31.91 ± 4.67^#^	41.31 ± 5.95^†#^

Values are presented as mean ± SD; *n* = 10.

*P* < 0.05 compared to ankle angle at: neutral (*); DF and neutral (†).

*P* < 0.05 compared to muscle state at: rest (§); rest and during MVC (#).

MG, medial gastrocnemius; DF, 20° dorsiflexion; PF, 30° plantar flexion; TMS, transcranial magnetic stimulation; MVC, maximum voluntary contraction.

#### Fascicle relaxation and MTJ displacement

**Figure 4:**
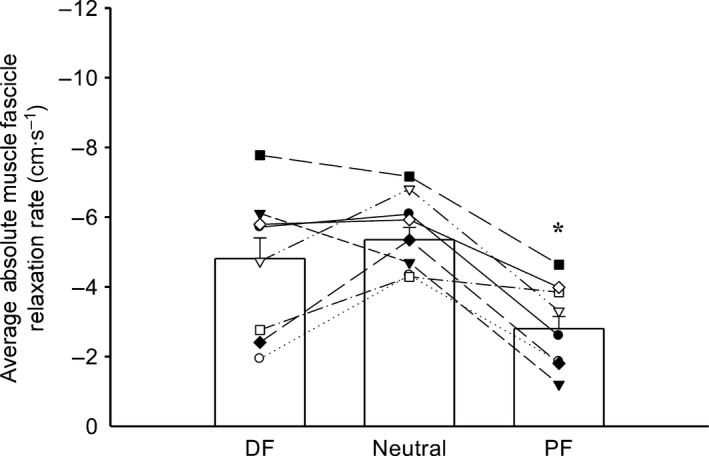
Effect of ankle angle (DF = 20° dorsiflexion, neutral, and PF = 30° plantar flexion) on average absolute MG muscle fascicle relaxation rate (*n* = 10). Columns represent group means ± SEM, whereas data points connected by lines indicate mean values for individual subjects. Muscle fascicle relaxation was slower for PF compared to DF and neutral positions (*P < 0.05).

**Figure 5:**
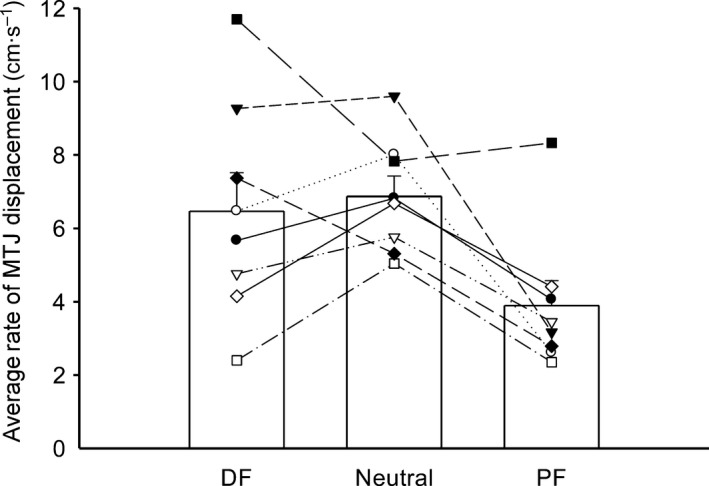
Effect of ankle angle (DF = 20° dorsiflexion, neutral, and PF = 30° plantar flexion) on average rate of muscle–tendon junction (MTJ) displacement (*n* = 8). Columns represent group means ± SEM, whereas data points connected by lines indicate mean values for individual subjects. There was a strong trend (*P* = 0.06) toward a main effect of angle on rate of MTJ displacement for PF compared to DF and neutral positions.

A one‐way repeated measures ANOVA failed to yield a main effect (*F*(2,16) = 2.570; *P* = 0.108) of ankle angle for average muscle fascicle relaxation time (0.29 ± 0.05 sec DF, 0.27 ± 0.06 sec neutral, and 0.28 ± 0.05 sec PF). However, there was a main effect (*F*(2,18) = 15.832; *P* < 0.001) of ankle angle for average absolute muscle fascicle relaxation rates. These rates were not significantly different between DF (−4.81 ± 1.87 cm·s^−1^) and neutral positions (−5.35 ± 1.10 cm·s^−1^; *P* = 0.813); but, both DF and neutral angles had significantly faster peak relaxation rates compared to PF (−2.80 ± 1.10 cm·s^−1^; *P* < 0.001 and *P* = 0.002, respectively; see Fig. 4). There was a trend (*F*(2,12) = 3.597; *P* = 0.060) toward an effect of angle on average rate of MTJ displacement (6.36 ± 2.97 cm·s^−1^ DF, lower bound 3.74 cm·s^−1^, upper bound 9.183 cm·s^−1^; 6.87 ± 1.55 cm·s^−1^ neutral, lower bound 5.562 cm·s^−1^, upper bound 8.181 cm·s^−1^; and 3.89 ± 1.93 cm·s^−1^ PF, lower bound 2.093 cm·s^−1^, upper bound 5.681 cm·s^−1^; see Fig. 5).

## Discussion

This study was designed to investigate the rate of torque and fascicle relaxation as well as MTJ displacement following high‐intensity TMS during maximal voluntary contractions at three muscle lengths. Our hypotheses were confirmed as muscle length influenced the relaxation rate of plantar flexor torque and MG fascicles. That is, absolute plantar flexor and MG fascicle relaxation rates were markedly slower when positioned at a shortened muscle length (30° PF) compared to longer lengths (neutral or 20° DF). The rate of MTJ displacement showed a similar trend (*P* = 0.06). The MG data show that TMS paired with ultrasound imaging is a feasible technique to obtain in vivo measurements of relaxation in muscle fascicles (and also the MTJ; Figs. 4 and 5).

### TMS‐induced relaxation technique

When TMS is delivered during an MVC, the interruption of voluntary drive (silent period) leads to a drop in torque which, as previously reported by McNeil et al. (2013), yields a measure of muscle relaxation rate that is functionally superior to the rate obtained with electrical stimulation delivered at rest. Although delivery of peripheral nerve stimulation during an MVC would elicit a short silent period, it would not completely inhibit ongoing EMG activity (the silent period would reflect collision of single orthodromic and antidromic impulses), while TMS is capable of the near‐complete abolishment of descending drive (Inghilleri et al. 1993).

Prior to the current experiment, few studies have investigated the effect of ankle angle (i.e., muscle length) on intrinsic contractile properties of the plantar flexor muscle group in humans (Sale et al. 1982; Mayfield et al. 2016a), of which only Sale and colleagues measured components of plantar flexor relaxation. Using electrical stimulation of the tibial nerve at rest, Sale and colleagues found half‐relaxation time to be slower at 20° DF (139.0 ± 26.5 ms) compared to 30° PF (89.5 ± 21.4 ms). Interestingly, our results for relaxation rate during an MVC are opposite, where 30° PF was found to have a significantly slower absolute rate of relaxation (−804 ± 162 Nm·s^−1^) compared to 20° DF (−2008 ± 692 Nm·s^−1^). This discrepancy is almost certainly caused by the abolition of the resting twitch when the ankle was moved from 20° DF to 30° PF (13.89 ± 0.75 vs. 0.54 ± 0.19 Nm, respectively; Sale et al. 1982). Such small twitches preclude meaningful insight into muscle relaxation, particularly when assessed using absolute time; that is, a twitch with an amplitude <4% of its comparator will unquestionably have a briefer half‐relaxation time. In the present study, MVC torque at 30° PF was still 34% of that at 20° DF and, despite the slower rate of relaxation, the absolute time of MG fascicle relaxation was virtually the same (0.29 ± 0.05 and 0.28 ± 0.05 sec for DF and PF, respectively). These data support TMS delivery during an MVC as the superior technique to assess muscle relaxation.

As with previous studies utilizing the TMS‐induced relaxation technique, relaxation rate was also expressed relative to the maximal torque prior to the silent period. In this study, normalized relaxation rates were not different across ankle angles (*P* = 0.312). This suggests that controlling for maximal torque at the time of stimulation normalizes for muscle length‐based differences in parameters, such as passive tension as well as cross‐bridge and joint mechanics.

### Muscle fascicles and MTJ

The most novel component of this study was the use of ultrasound imaging in conjunction with TMS. We found ultrasound imaging technology to be capable of capturing TMS‐induced relaxation in MG fascicles (Fig. 3) and MTJ displacement (Fig. 2). Moreover, it successfully detected the influence of muscle length on the relaxation rate of MG fascicles. As expected, and documented previously (e.g., Ito et al. 1998; Arampatzis et al. 2007), we found significant decreases in MG fascicle length and increases in pennation angle during the transition from rest to MVC. We extend these findings to show that fascicle lengths during the TMS‐induced silent period lie between the extremes of MVC and rest (Table 2). Fascicles did not lengthen to their initial resting value because participants were instructed to redevelop torque as soon as possible after the stimulus, which enabled voluntary drive to be re‐established (the silent period ended) before the muscle had relaxed completely (see Fig. 1). The absolute time (in milliseconds) for relaxation of MG fascicles was not significantly different across ankle angles (*P* = 0.108). This provides evidence that descending drive from the primary motor cortex, which is transiently interrupted by TMS (the silent period), recovers within the same time frame, independent of ankle angle. Therefore, any significant difference among relaxation rates at the three ankle angles cannot be due to variability in the recovery time of descending drive. This suggests that the significantly slower absolute relaxation rates observed in the plantar flexed position are determined primarily by the length–tension relationship of the muscle (see below) and compliance of the series‐elastic element (SEE).

### Length‐dependent mechanisms potentially influencing relaxation rate

In addition to extensive foundational basic research (e.g., Hill 1970), recent evidence supports a profound impact of SEE compliance (manipulated by tendon stiffness) on rate of torque development in humans (Mayfield et al. 2016a) and relaxation rate in isolated cane toad muscle (Mayfield et al. 2016b). Our data provide in vivo evidence for the impact of muscle length‐dependent passive tension on intrinsic contractile properties as we show a significant increase from PF to DF which explained ~24% (*r* = 0.493) of the variation seen in the absolute plantar flexor relaxation rate.

Although the techniques of the present experiment do not permit speculation on the impact such mechanisms might have on our relaxation rate data, in vitro research has shown that calcium kinetics (Stephenson & Wendt 1984) and sarcomere dynamics (Edman & Flitney 1982) are length dependent. With respect to sarcomere dynamics, it is worth noting that sarcomeres of the MG in humans are estimated to operate only on the ascending limb of the force–length curve (Maganaris 2003). Similarly, the sarcomeres of the soleus are estimated to be confined to the ascending limb and plateau portion of the force–length curve (Maganaris 2001).

In addition to the length–tension relationship, the potential contributions of neural mechanisms must also be considered as knee angle impacts surface (Kennedy and Cresswell 2001; McNeil et al. 2013) and intramuscular EMG (Kennedy and Cresswell 2001) of the plantar flexors. In our study, voluntary activation estimates were >97% at all ankle angles, so inadequate descending drive is unlikely to contribute to the slower relaxation rates at shorter muscle lengths.

In conclusion, the results support our hypothesis, such that: (1) changes in muscle length do significantly impact the absolute plantar flexor relaxation rate induced by TMS; (2) in vivo changes of muscle architecture were successfully captured using ultrasound technology and varied with joint angle in a manner similar to the torque data. These results indicate that TMS and ultrasonography can be paired to investigate the intrinsic contractile properties of muscle fascicles in vivo in the presence of descending drive, increasing our ability to measure muscle function in a more functionally relevant state than previous assessments employing electrical stimulation to a relaxed muscle.

## Conflict of Interest

The authors declare they have no conflict of interest.
